# Moral judgment modulation by disgust is bi-directionally moderated by individual sensitivity

**DOI:** 10.3389/fpsyg.2014.00194

**Published:** 2014-03-06

**Authors:** How Hwee Ong, O’Dhaniel A. Mullette-Gillman, Kenneth Kwok, Julian Lim

**Affiliations:** ^1^Department of Psychology, National University of SingaporeSingapore, Singapore; ^2^Neuroscience and Behavioral Disorders, Duke-NUS Graduate Medical SchoolSingapore, Singapore; ^3^Neurobiology Programme, National University of SingaporeSingapore, Singapore; ^4^Singapore Institute for Neurotechnology, Centre for Life Sciences, National University of SingaporeSingapore, Singapore; ^5^Cognitive Science Lab, Temasek Laboratories, National University of SingaporeSingapore, Singapore

**Keywords:** moral judgment, disgust, subliminal priming, utilitarian, decision-making

## Abstract

Modern theories of moral judgment predict that both conscious reasoning and unconscious emotional influences affect the way people decide about right and wrong. In a series of experiments, we tested the effect of subliminal and conscious priming of disgust facial expressions on moral dilemmas. “Trolley-car”-type scenarios were used, with subjects rating how acceptable they found the utilitarian course of action to be. On average, subliminal priming of disgust facial expressions resulted in higher rates of utilitarian judgments compared to neutral facial expressions. Further, in replication, we found that individual change in moral acceptability ratings due to disgust priming was modulated by individual sensitivity to disgust, revealing a bi-directional function. Our second replication extended this result to show that the function held for both subliminally and consciously presented stimuli. Combined across these experiments, we show a reliable bi-directional function, with presentation of disgust expression primes to individuals with higher disgust sensitivity resulting in more utilitarian judgments (i.e., number-based) and presentations to individuals with lower sensitivity resulting in more deontological judgments (i.e., rules-based). Our results may reconcile previous conflicting reports of disgust modulation of moral judgment by modeling how individual sensitivity to disgust determines the direction and degree of this effect.

## INTRODUCTION

Moral conflict arises when an individual has the option of bringing about the greater good by taking an emotionally unappealing course of action. While a lot of individuals might readily endorse the “Spock principle” – that the needs of the many outweigh the needs of the few – there are perhaps equally many who would make the reverse decision in real life, for example, declining to donate money even when told categorically that withholding action would cost lives (Unger, 1996). It is thus important to understand the factors that affect people’s moral decisions in order to help individuals and societies maximize the outcomes that arise from them.

A classic laboratory illustration of a moral dilemma is the trolley-car scenario, in which one has the option of pushing a large person in front of an on-rushing trolley in order to save the lives of five people who are ahead of it on the tracks. In philosophical terms, such a dilemma can be thought of as a decision between a deontological or utilitarian course of action; that is, abiding by a principle (e.g., “I should not actively cause harm to another person”) versus maximizing utility (e.g., saving the most lives). Situations such as the trolley-car dilemma have been used increasingly in psychological studies as a means of isolating the components of the moral decision-making process, and in elucidating the situational factors that may influence people’s choices.

Among the known factors that alter this decision profile is emotional state. Modern theories of decision-making have emphasized that emotions may play a significant role in this process, accounting for deviations from rationalist or normative models (Mullette-Gillman et al., 2011). Two such psychological theories of moral decision-making that have arisen in the past decades are the social intuitionist theory of Haidt (2001), and the dual process theory of Greene et al. (2001, 2004), Paxton and Greene (2010). Though these theories differ in certain particulars, both posit that emotions may interact with, or in some cases overrule rational processes in moral decision-making. Thus, in trolley-car-type dilemmas, subjects putatively resist the utilitarian option due to visceral feelings of arousal, fear or disgust that are triggered by thoughts of having to bring about personal harm.

In the current work, we began by investigating the effect of subliminal disgust induction on moral decision-making. Disgust was the focus of study in this context due to its *a priori* link with moral transgressions, particularly when standards of purity are breached (Rozin et al., 1999; Horberg et al., 2009). Accordingly, disgust primes have been found to cause subjects to make harsher judgments of morally unacceptable actions (Wheatley and Haidt, 2005; Schnall et al., 2008b). However, effects on moral dilemmas (as opposed to judgments of moral wrongness) have not been demonstrated with unconscious emotional cues. Showing an effect of subliminal priming on moral dilemmas would strengthen the case for the dual process theory by diminishing the possibility of the primes having a direct effect on conscious, pre-decision thoughts and reasoning. In other words, conscious primes may affect rational deliberation through either conscious or subconscious processes, whereas subliminal primes are more likely to affect decisional outcomes via unconscious processes alone. In the current context, we define moral intuition as the rapid, non-reflective process whereby certain moral conclusions are reached and regarded as self-evident (Haidt, 2001).

Based on previous experiments, we hypothesized that subliminal disgust priming would lead subjects to rate utilitarian actions as less morally acceptable (i.e., a shift toward more deontological, or principled, selections). This hypothesis was based on several prior studies showing that disgust primes increase the severity of judgments when subjects were asked to rate moral transgressions. These studies utilized manipulations such as the hypnotic induction of disgust (Wheatley and Haidt, 2005), disgusting smells (Schnall et al., 2008b; Ugazio et al., 2012), and a gustatory disgust prime (a bitter beverage; Eskine et al., 2011). Interestingly, reversing the valence of the manipulation appears to have the opposite effect – Schnall et al. (2008a) showed that priming individuals with cleanliness concepts reduced the severity of their moral ratings on similar vignettes.

We tested this hypothesis by priming subjects with subliminally presented emotional faces, with forward and backward masking, followed by presentation of the same face with a neutral expression, a method that has previously been shown to effectively influence behavior without affecting conscious emotional state (Nomura et al., 2004; Winkielman et al., 2005). Here, we report the results of two experiments designed to test for priming effects under differing conditions in order to gain a better understanding of the relationship between disgust and moral decision-making. In brief, we find that disgust priming modulates utilitarian decision-making, with an average enhancement of utilitarian judgment, but that the direction and degree of modulation depends on individual sensitivity to disgust.

## EXPERIMENT 1

### METHOD

#### Participants

Experiment 1 was composed of two independent samples of undergraduates from the National University of Singapore. Participation was restricted only to Singaporeans in order to control for potential cross-cultural variability in moral thinking. Sample A contained 23 participants [11 females; mean age = 22.30 (SD = 1.36)] and sample B contained 28 participants [18 females; mean age = 21.68 (SD = 1.47)]. Two subjects from sample A and one subject from sample B were excluded from analysis due to technical or experimenter errors. Participants received either course credit or monetary compensation for their time. All experiments were approved by the Institutional Review Board of the National University of Singapore, and participants gave written informed consent before taking part in the study.

#### Moral dilemmas

**FIGURE 1:**
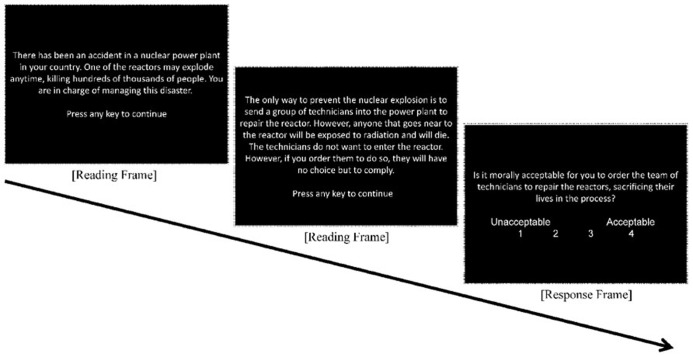
**Example of a complete moral dilemma (broken into three screens as subjects would view it)**.

Across the two experiments, subjects were presented with a series of moral dilemmas and asked to judge the moral acceptability of performing the described utilitarian option (**Figure 1**). Each dilemma was composed of a short vignette leading to a crisis, followed by an action that could resolve the crisis. Subjects were asked to rate how morally acceptable it was to perform the described action. These questions were all framed such that performing the action would result in the most utilitarian final outcome by causing harm to an individual.

Fifty-one dilemmas were either created or adapted from prior studies by Greene et al. (2001, 2004) and internet sources (Listverse, 2007; Akorra.com, 2010; Hopkins, 2011). Dilemmas were edited for word length and comprehensibility. An example dilemma is presented within **Figure 1**.

A pilot study was conducted to estimate normative acceptability ratings for each of these dilemmas. *N* = 63 subjects rated the moral acceptability of the 51 vignettes on a 5-point Likert-scale (1 = *Unacceptable*, 5 = *Acceptable*). Across dilemmas, the mean (SD) subject rating was 2.46 (0.88). As our goal was to examine the decision-making, we selected the central 40 dilemmas, removing the 11 dilemmas with the most extreme average acceptability ratings (mean acceptability below 1.54 or above 4.23) that could produce floor or ceiling effects. These dilemmas were then split into two lists of 20 stimuli each, with similar acceptability ratings [mean(SD) = 2.67(0.70) and 2.68(0.71)].

#### Procedure

**FIGURE 2:**
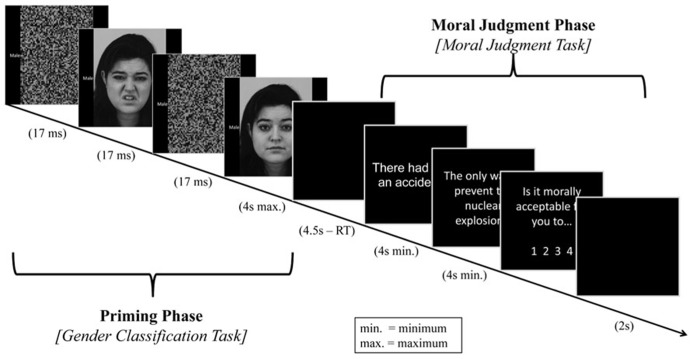
**Schematic of one experimental trial (priming phase + moral judgment phase).** The face shown here is M31DI from the Karolinska Directed Emotional Faces (KDEF) stimulus set.

Study data were collected in the experimental suites of the Cognitive Science Lab in Temasek Laboratories and in the Psychology Department labs in the National University of Singapore. Data were presented on Windows computers via E-Prime 1.1 (Schneider et al., 2002). Each trial consisted of two phases, a priming phase and a moral judgment phase. **Figure 2** depicts the stimulus presentation order in each trial. The procedures of the priming phase were adapted from an existing study (Winkielman et al., 2005). Images of emotional facial expressions were adapted from the Karolinska Directed Emotional Faces (KDEF) database (Lundqvist et al., 1998). All images were converted to gray-scale and overall luminosity was controlled. Pattern masks were created by using Adobe Photoshop CS6 to scramble similarly processed face stimuli (Yang et al., 2011). Face stimuli that were used to create the mask were not used in other parts of the experiment.

During the priming phase of each trial, participants were first presented with a forward pattern mask (16.66 ms), followed by a subliminal facial prime (16.66 ms), then a backward pattern mask (16.66 ms). A target face with the words “Male” and “Female” on its left and right side respectively then replaced the final mask, and participants were required to classify the gender of this target through a keyboard press. The target face remained until the participant made a response (4 s max). Within each trial, the prime and target face always belonged to the same person. During the moral judgment phase, participants read and made a judgment on a moral dilemma. The text of each dilemma was divided into three screens (illustrated in **Figure 1**); the first two screens consisted of the scenario description, while the third screen asked participants to rate the acceptability of the described utilitarian action. To encourage a thorough reading of the dilemmas, participants had to wait a minimum of 4s on each of the first two reading screens before they were allowed to proceed.

Before starting the actual tasks, participants performed a practice session of three trials to familiarize themselves with the format of the dilemmas and responses. These trials consisted of moral decisions that did not involve personal harm.

Participants then completed two blocks of 20 moral dilemmas each (total of 40). Subliminal primes were images of disgusted facial expressions in one block (i.e., disgust block), and images of neutral facial expressions in the other block (i.e., neutral block). The order of the blocks and the moral dilemmas used within each block were counter-balanced across participants. To reduce carry-over effects, the two blocks were separated by a 7-minute interval, during which participants watched a video clip of a documentary about coral reefs. At the beginning and end of each block, participants rated their current emotional state on four dimensions (disgust, happiness, anger and sadness), of which disgust was our scale of interest. Responses were collected on a 0 to 10-point scale, with 0 being “not angry/disgusted/happy/sad at all” and 10 being “very angry/disgusted/happy/sad”.

Finally, subjects were debriefed and asked to guess the purpose of the experiment. They were specifically asked if they had detected any subliminal primes before being debriefed on the true purpose of the study. None of the participants reported detecting the primes or guessed the study aims.

Subjects in sample B underwent an identical procedure to those in Sample A, with the addition of completing the Disgust Scale – Revised [DS-R; (Haidt et al., 1994), modified by Olatunji et al. (2007)]. We added this scale with the hypothesis that disgust sensitivity would moderate the modulation of moral judgment by disgust priming observed in Sample A.

## RESULTS

### SAMPLE A

Mean acceptability ratings were higher in the disgust condition (*M* = 2.59, SD = 0.52) than the neutral condition (*M* = 2.40, SD = 0.37), *t*(22) = 3.03, *p* < 0.01, *d* = 0.63.

As this effect was in the opposite direction of our anticipated effect, based upon prior studies (Wheatley and Haidt, 2005; Schnall et al., 2008b), we immediately performed a replication in an independent sample. The sole change between the two experiments was the addition of the Disgust Scale – Revised [DS-R; (Haidt et al., 1994), modified by Olatunji et al. (2007)] at the end of the experimental session.

### SAMPLE B

**FIGURE 3:**
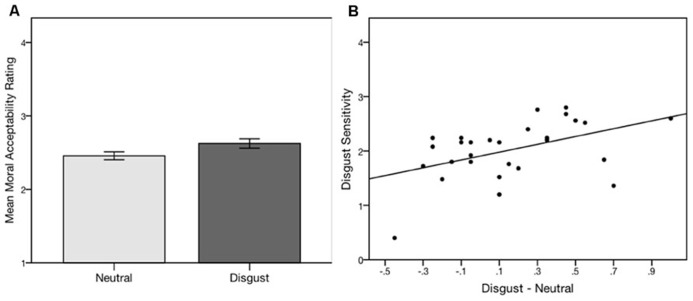
**(A)** Subliminal disgust priming followed by self-paced screen presentations of moral dilemmas caused subjects to make more utilitarian responses. Error bars represent +/- 1 SE. **(B)** The degree of influence of the prime (as measured by the difference in mean response in the disgust and neutral conditions) is moderated by disgust sensitivity. Note the bi-directional function, with increased acceptability (more utilitarian) ratings for individuals with higher sensitivity and decreased acceptability for individuals with lower sensitivity.

The replication sample yielded similar results: the mean acceptability rating in the disgust condition (*M* = 2.65, SD = 0.40) was higher than that in the neutral condition [*M* = 2.50, SD = 0.40; *t*(27) = 2.36,* p* < 0.05, *d* = 0.45]. Combining samples A and B yielded a highly significant main effect [disgust: *M* = 2.63, SD**= 0.45; neutral: (*M *= 2.46, SD = 0.39), *t*(50) = 3.75, *p* < 0.001, *d* = 0.53 (**Figure 3A**)].

To test whether this change represented a switch in the valence (as opposed to the intensity) of subjects’ responses, we collapsed the 4-point responses into two bins: “acceptable” and “unacceptable.” We found that the mean proportion of questions to which subjects responded “acceptable” was significantly higher in the disgust group (56.47%) than the neutral group (49.80%), *t*(50) = 3.20, *p* < 0.01, *d* = 0.45.

Difference scores were then obtained for each participant by subtracting the mean acceptability ratings in the neutral condition from the disgust condition. A positive difference score indicates higher acceptability ratings in the disgust relative to the neutral condition. These scores were positively correlated with DS-R scores in the replication sample (*r* = 0.47, *p* < 0.05; **Figure 3B**). A one-way repeated-measures analysis of covariance (ANCOVA) between the disgust and neutral conditions with DS-R as a covariate was just above the threshold of statistical significance, [*F*(1,26) = 3.87, *p* = 0.06, $\eta_{partial}^{2\;\;\;\;\;\;}$ = 0.13] (moderate effect size; Richardson, 2011).

We tested for differences in participants’ self-reported level of disgust by performing one-sided *t*-tests of the change in rating against 0. Disgust ratings were significantly increased between the beginning and end of both disgust [*t*(50) = 5.94, *p* < 0.001] and neutral blocks [*t*(50) = 5.44, *p* < 0.001], with no significant difference in the change between these blocks [disgust: *M* = 1.16, SD = 1.39; neutral (*M* = 1.18, SD = 1.55), *t*(50) = 0.10, *n.s.*]. The change in self-reported disgust over the disgust block was not significantly correlated with DS-R (*r* = 0.11, *n.s.*).

Our analysis was focused on the emotion of disgust, given our use of disgust expressions and the prior literature’s focus on disgust. However, as we also collected information that could reveal state alterations in three other emotions (anger, sadness, and happiness), we also tested for manipulation-related alterations in these. Interestingly, we found significant increases across all blocks in anger [Disgust: *t*(50) = 3.79, *p* < 0.001; Neutral: *t*(50) = 3.19, *p* < 0.05] and sadness [Disgust:* t*(50) = 4.09, *p* < 0.001; Neutral: *t*(50) = 4.02, *p* < 0.001] and a significant decrease in happiness [Disgust: *t*(50) = -5.59, *p* < 0.001; Neutral: *t*(50) = -5.08, *p* < 0.001]. There was no significant difference in the changes of these other emotions between the disgust and neutral blocks.

A 2x2 between-subject analysis of variance (ANOVA) was conducted to test for differences between the counter-balancing conditions. We found no significant interaction between the two counterbalanced Lists and Orders, [*F*(1,47) = 0.58, *n.s.*], and no main effects of List, [*F*(1,47) = 0.76, *n.s.*], or Order, [*F*(1,47) = 0.21, *n.s.*]

As the results obtained in Experiment 1 ran counter to the direction found in most previous published experiments, we ran a second experiment using conscious primes, which were used by a majority of previous studies, to see if the same pattern of results would hold.

## EXPERIMENT 2

### METHOD

#### Participants

Participants were 26 undergraduates (16 females) at the National University of Singapore. Participants received monetary compensation. Their mean age was 21.73 (SD**= 1.71). Participation was restricted to Singaporeans to control for cross-cultural variability.

#### Procedure

In contrast to Experiment 1, in Experiment 2 the emotional primes were presented consciously. This difference occurred during the priming phase, during which participants were first presented with an image of a face for 2–3 s (jittered). The word “Male” and “Female” then appeared on the left and right side of the face respectively, cueing participants to classify the gender of the face, with a maximum of 1 s to make their response. This altered priming phase was then followed by the moral judgment phase, which was unaltered from Experiment 1.

## RESULTS

**FIGURE 4:**
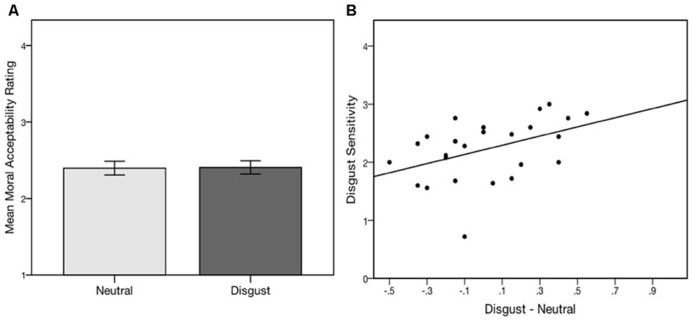
**(A)** Conscious disgust priming followed by self-paced screen presentations of moral dilemmas had no main effect on moral judgments. Error bars represent +/- 1 SE. **(B)** The degree of influence of the conscious prime (as measured by the difference in mean response in the disgust and neutral conditions) is moderated by disgust sensitivity. Note the bi-directional function, with increased acceptability (more utilitarian) ratings for individuals with higher sensitivity and decreased acceptability for individuals with lower sensitivity.

Using conscious priming, we found no significant main effect of emotion on moral acceptability between the disgust condition (**Figure 4A**; *M* = 2.41, SD = 0.44) and neutral condition (*M* = 2.40, SD = 0.46), [*t*(25) = 1.71, *n.s*]. However, difference scores were once again positively correlated with individual disgust sensitivity (DS-R) scores in this sample (*r *= 0.43, *p* < 0.05; **Figure 4B**), and the one-way repeated-measures ANCOVA with DS-R as a covariate was significant, *F*(1,24) = 5.08, *p* < 0.05, $\eta_{partial}^{2\;\;\;\;\;\;}$ = 0.18 (moderate effect size; Richardson, 2011), suggesting that DS-R was still a moderator of the priming effect.

As in Experiment 1, self-reported disgust increased significantly from the beginning to the end of both the disgust [*t*(25) = 4.48, *p* < 0.001] and neutral blocks [*t*(25) = 2.72, *p* < 0.05]. These change in these levels was not significantly different between the disgust block (*M* = 1.03, SD = 1.18) and the neutral block (*M* = 0.96, SD = 1.80), [*t*(25) = 0.22, *n.s.*]. Again, there was no correlation between DS-R and self-reported increases in disgust in the block with the disgust primes (*r* = 0.10, *n.s.*).

Certain non-disgust emotions were also significantly altered by this paradigm. Subjects felt significantly less happy after the disgust block [*t*(25) = -2.95, *p* < 0.05], and significantly sadder after both blocks [Disgust: *t*(25) = 2.162, *p* < 0.001; Neutral: *t*(25) = 2.10, *p* < 0.001]. There was no significant difference in the changes of these other emotions between the disgust and neutral blocks.

A 2x2 between-subject ANOVA was conducted to test for differences between the counter-balancing conditions. We found no significant interaction between the two counterbalancing Lists and Orders, [*F*(1,22) = 3.56, *n.s.*], and no main effects of List [*F*(1,22) = 0.55, *n.s.*], or Order, [*F*(1,22) = 0.44, *n.s.*].

## COMBINED ANALYSIS

To test the effects of subliminal vs. conscious priming, we combining the data from experiments 1b and 2 into a single model with change in acceptability rating as a dependent variable, type of priming as a factor, and DS-R as a covariate. We found a significant effect of DS-R, [*F*(1,50) = 12.67], *p* = 0.001, no effect of priming type, [*F*(1,50) = 0.29, *n.s.*], and no significant interaction [*F*(1,50) = 0.194, n.s.].

## DISCUSSION

We find that the moral acceptability of a utilitarian action is bi-directionally modulated by disgust facial expressions as a function of individual sensitivity to disgust. This function was found across both subliminal and conscious presentation of disgust facial expressions. This result clearly shows that individual appraisal of the stimuli determines both the direction and size of judgment modulation.

Our original experimental hypothesis was that subliminal disgust priming would decrease the acceptability of utilitarian actions in trolley-car type moral dilemmas. We did not find evidence in support of this. Rather, our results suggest an explanation for the conflicting findings across previous studies. We discuss the possibility that rather than acting directly through induced disgust emotions, the disgust primes may alter moral judgment by influencing social processing.

## DISGUST PRIMING MODULATES MORAL JUDGMENT

Rational models of decision-making assert that alternatives are weighed based on the expected value of outcomes, and place little or no emphasis on emotional processes. Our data add to a growing body of work that demonstrates that emotional information is regularly integrated in decision-making. Within the realm of moral decision-making, the dual process theory of Greene et al. (2001, 2004), Paxton and Greene (2010) has been one of the leading attempts to formalize this. Their theory proposes that deontological and utilitarian judgments are driven by dissociable cognitive systems, and that subjects tend to make more utilitarian judgments when they engage in controlled, rational processing, and more deontological judgments when using intuitions or emotion. Specific emotional states can thus be incorporated into the decision-making process, causing subjects to deviate from their baseline profile of responding. 

Superficially, the effects found in the current paradigm run counter to our *a priori *hypothesis that disgust facial expressions would result in decreased moral acceptability for the utilitarian action. Unfortunately, two recent studies have suggested that the effects of disgust-priming on moral decision-making are not so simple. Cummins and Cummins (2012) reported that exposure to positive visual stimuli increased the number of deontological decisions selected by subjects, and La Rosa et al. (2011) found that priming with disgusting images resulted in less severe judgments in such moral dilemmas. In summary, although a larger number of published studies to date have linked disgust priming to harsher moral judgments and more deontological choices, there have also been multiple demonstrations of effects in the opposite direction.

Our current data suggest a potential explanation for these mixed findings: the effect of disgust priming on moral judgments is moderated by how sensitive the individual is to disgust stimuli. Thus, in samples where average disgust sensitivity is relatively low, the average effect of disgust priming would be to decrease levels of utilitarian responding (lower acceptability), potentially resulting in the disgust-induced harsher judgments found in early publications (Wheatley and Haidt, 2005; Schnall et al., 2008b; Eskine et al., 2011; Ugazio et al., 2012). Samples with “average” levels of disgust sensitivity would lead to no average effect (as we see in Experiment 2, and which may have occurred in numerous experiments which went unpublished). Finally, samples with participants high in disgust sensitivity (as in Experiment 1B, and presumably 1A), would average an increase in utilitarian judgments (higher acceptability ratings), which might also account for the results of multiple recent studies (La Rosa et al., 2011; Cummins and Cummins, 2012).

With the current dataset, we cannot make any definitive claims about the mechanisms that underlie the modulatory effect of disgust sensitivity. We discuss one potential candidate – sociality – in the following section. However, further work using neuroimaging or different behavioral paradigms is necessary to answer this open question.

## ROLE OF SOCIALITY IN MORAL DECISION-MAKING

Although disgust sensitivity moderates the effects of disgust priming, the psychological mechanisms responsible for altering an individual’s decisions may not be the actual induction of disgust. This is supported by the fact that while we did find an increase in subjective disgust ratings over the disgust block, the change in state did not correlate with changes in acceptability ratings or disgust sensitivity, and was also found over the neutral block.

We speculate that, rather than inducing disgust emotions, the disgust primes may alter moral judgment by influencing social processing, possibly through altering individual prosocial preferences. Supporting this theory, it has been demonstrated that dehumanization of the person to be sacrificed in the dilemma can cause subjects to choose the utilitarian option more frequently (Majdandzic et al., 2012), and disgust, in turn, has been shown to facilitate dehumanization (Hodson and Costello, 2007; Buckels and Trapnell, 2013).

Sociality may come into play to a much greater degree in our study because of our choice of disgust facial expressions as a prime, as opposed to disgusting environments or scenes. Wild et al. (2001) theorized that emotional contagion from viewing the expressions of conspecifics may be a “prewired” mechanism that is crucial for effective social interaction, whereas aversion to disgusting pictures or environments may not have the same degree of social relevance. Inducing emotions by observing conspecifics may thus be different from viewing a scene or appraising an environment due to these additional social cues. In support of this, numerous studies have shown differential neural processing for faces and scenes (Sabatinelli et al., 2011). Importantly, face processing regions (anterior fusiform gyrus and middle temporal gyrus), are involved in processing emotional face stimuli even after subtraction of activation to neutral faces to account for perceptual processes. Testing the paths from disgust to sociality to moral decisions in a single study is thus a logical next step in this area of research.

## SUBLIMINAL vs. CONSCIOUS PRIMING

With disgust sensitivity as a covariate in our model, both subliminal and conscious disgust primes altered moral judgments. This strengthens the case for the dual process model (Greene et al., 2001) by showing that information presented outside of conscious awareness can still be factored into moral judgments and decisions. We also corroborate the findings of other researchers who have reported subliminal priming effects for moral rules (Broeders et al., 2011), and religious primes (Saraglou et al., 2009) on subsequent moral behavior.

Previous studies have suggested that subliminal emotional primes have a stronger effect than supraliminal primes on subjects’ subjective ratings of subsequently presented stimuli (Murphy and Zajonc, 1993; Rotteveel et al., 2001). These authors argued that emotional information from subliminal primes travels via a more direct neural pathway to be integrated in decisions and output than information from conscious primes. Indeed, several fMRI studies have found evidence for differences in the neural processing of subliminal and supraliminal stimuli (Dehaene et al., 2001; Kouider et al., 2007).

Within our study, given the mean difference in moral judgment in experiment 1 (subliminal) and the lack of a mean difference in experiment 2 (conscious primes), it could be tempting to conclude that the effects of subliminal priming were stronger than that of supraliminal priming. However, when individual sensitivity is taken into account, presentation threshold (subliminal vs. conscious) did not moderate the effects of disgust priming on moral judgments. This highlights the importance of using individual sensitivity as a covariate before interrogating the effects of other experimental variables in future studies of emotion and moral judgment. The domain-specificity of differences between subliminal and conscious remains to be explored, and our present findings may not generalize to priming studies as a whole.

## CHANGES IN NON-DISGUST EMOTIONS

Besides disgust, we also collected subjective ratings of three other emotions (happiness, sadness, and anger) before and after each block of dilemmas. Interestingly, we found increases in anger and sadness and decreases in happiness on all task blocks in Experiment 1, and moderate changes in many of these emotions in Experiment 2. These data suggest that the act of deliberating trolley-car type moral dilemmas on its own may be sufficient to induce a range of negative-valenced emotions. We also speculate that variance associated with these changes may have masked any increase in disgust that was specific to the priming effect in our current experiment. Further research could be performed to disentangle the effects of disgust on moral decision-making from those of negative-valenced emotions more generally.

## CULTURAL DIFFERENCES

Our experiment is the first to test the effects of emotional priming on moral dilemmas in an Asian population, raising the issue of cultural differences with other published work. Holding other variables constant, Moore et al. (2011) found that college students in the USA and Hong Kong did not differ significantly in their response patterns to personal and impersonal moral dilemmas, suggesting that baseline responding alone did not account for our findings. However, there is some evidence that triggers of disgust may differ based on cultural norms (Haidt et al., 1993) or political attitudes (Inbar et al., 2009). Replication of the current finding in a Western population is thus needed to definitively disconfirm this alternative hypothesis.

## CONCLUSION

In the current set of studies, we present evidence that disgust sensitivity is a moderator of the effect of disgust priming on moral judgments. These data complement the body of evidence showing that both reason and emotion play a part in guiding moral judgment while offering an explanation for the contradictory results in this literature.

The subtlety of the priming used in this experiment coupled with its relatively large effect sizes lead us to speculate that these effects may not just be phenomena observed in the laboratory, but may reflect responses to social cues (e.g., microexpressions) in real-world situations. If so, it may be incumbent on us to take extra care when making moral judgments to ensure that reason truly prevails.

## AUTHOR CONTRIBUTIONS

All authors contributed to designing the experiment and analyses, as well as interpreting the results and writing the manuscript. How Hwee Ong and Julian Lim collected the data and performed the analyses.

## Conflict of Interest Statement

The authors declare that the research was conducted in the absence of any commercial or financial relationships that could be construed as a potential conflict of interest.
